# New Things in Dental Pathology

**Published:** 1914-11-15

**Authors:** O. E. Inglis


### NEW THINGS IN DENTAL PATHOLOGY

O. E. INGLIS, D.D.S.

To discuss all the new ideas within twenty minutes is impossible,. therefore I confine myself to something of immediate interest and propose taking up the microbic plaque. As ordinarily understood a microbic plaque is first a precipitation of mucin the sticky substance or solid matter in solution in the saliva. The teeth, plates, etc., being comparatively inert objects bathed in the saliva are always coated with a film of saliva and under conditions of rest as at night the mucin is deposited upon them and becomes infected with bacteria, which ferment it and make it still more adhesive. New deposits occur and then the film thickens. Tiny particles of food are caught in it and those are fermented by the bacteria. If these bacteria are of an acid producing variety and the food is of a nature fermentable into acid, such as carbohydrates, acid is formed, particularly latic acid which produces in suitable places the decalcification which is the first stage of dental caries.

If the bacteria are not acid producers or the food caught is not carbohydrate then an alkaline reaction is probably produced. It is well known that albuminoid materials thus ferment alkaline.

If the plaques contain pus organisms these act upon the gum structures and irritate them, producing inflammation and gum recession. No doubt some organisms aid the precipitation of the lime salts in the saliva into the previously formed plaque where they combine chemically with the albuminoid materials to form calculus. The movement of the teeth cause the sharp crystals to slightly rub the gum and mechanically increases the irritation by the bacteria or their products.

This in a nutshell is the cause of all the diseases of the teeth and gums which are not purely mechanical, such as abrasion or purely chemical or chemico-mechanical as erosion.

Practicall all the diseases associated with the teeth are the result of caries or pyorrhoea, and follow them, other infections being added after these results are produced. We may possibly except the diseases of dentition which are in the beginning mechanical disorders within the structures around the teeth. The microbic plaque then is the foundation of dental disease and as such merits our profound attention.

The removal of the plaque is then the indication and is the basis of prophylaxis. Can the plaque be removed and kept away? Probably not. Why then does prophylaxis every one, two or three months together with proper daily care by the patient prevent these two important diseases?

Let us reason by analogy. Certified milk commissions admit that 10,000 bacteria to the cubic centimeter in milk are practically harmless, while 500,000 become a source of great danger. This admits that numbers constitute a great factor, though, of course the 10,000 may not be typhoid or such pathogenic bacteria. If now we consider the plaque as removed frequently and of course frequently formed again, we must consider that the benefits of occasional prophylaxis arise from the fact, almost universal in plant life, that frequent disturbances of the course of nutrition inhibits growth.

Let us take a tree for example.

Frequent transplantation into new soil or in case of grown trees even one removal may cause the tree to die. The tree requires time to get set in the new location, to adjust its nutritive mechanism.

If grown grass is continually walked on growth is prevented.

We may look at the plaque in like manner. The plaque requires at least some time, just how much can not now be said, to become fixed and the dominant organisms to produce characteristic detrimental effects on tooth or gum.

There is a decided difference in action between bacteria implanted in living tissue and those simply resting against it. The effects of pus organisms are probably slower against the gum margin than in apical tissue. In the latter situation they act acutely, though sometimes chronically, in the former often chronically.

If now we rub off the plaque frequently the diseases do not occur. We even often cure established disease such as pyorrhoea by simply removing the cause (plaques) and if this is true we can easily prevent the disease altogether. Why then since people cleanse their teeth do we still have the disease in rich and poor alike. The answer is given by clinical evidence which is abundant.

Recently a patient returned after six months absence for a prophylaxis. She is scrupulous about her mouth, a brush and floss enthusiast, first because she has teeth of poor quality and at least fifty fillings large and small, the result of previous attacks of caries and repairs of the same, and she neither likes the loss of her teeth nor the work incidental to repair. Second she is rational and receptive to instruction and she sees that her care and mine have reduced her work to a joke, namely, one or two tiny new cavities or repairs and a cleansing, or even at times only a cleansing each six months. Yet with all her care I recently pointed out to her a few places at which her care was inoperative. She failed to brush the crevical half of her third molars. She did not carry her brush cervially but occlusally as she thrust it back at that point. There was no caries but in time that might have come.

From being a marked susceptible this patient has become almost an immune, and since her previous visit has even been through typhoid fever, which is a notorious caries breeder, without any effect upon her teeth.

Now we have all grades of cleanliness, or rather uncleanliness, and from the time of the introduction of the tooth brush dentists have had abundance of work. Why? Simply because partial cleanliness is nearly as bad and often worse than none.

Why should we think caries is due to systemic causes when obvious causes are under our eyes and can be, yet are not removed. Instruct a patient to thoroughly cleanse the teeth before coming to the office. Take equal parts of tincture of iodine and alcohol, and paint the teeth with it. Rinse off with water. If the teeth are stained plaques are present and these can be scraped off with an instrument while the patient views the operation in a mirror. Also at this time the action of floss or special instruments can be demonstrated.

Will the patient then use these implements intelligently thereafter? Perhaps so, if they are interested and desirous of good teeth. Perhaps not, if they are interested and lazy or think they are too busy with other things. Probably not if they half or wholly do not believe you even with their eyes on the plaques, *but* you will have done *your* duty and the rest is their affair.

There are only a few uses of the tooth brush:

1. To cleanse vigorously the fissures by strong occlusal brushing. Here failure is relatively liable owing to fissure depth.

2. To cleanse the cervical margins by a light to and fro motion *at the gum* not away from it. This can be fairly successful.

3. To cleanse with a rotary motion at the embrassures to remove food masses. This is relatively successful.

Teeth decay frequently in the neighborhood of the approximal contact point.

Here floss must be used and should be wrapped half around the tooth after being placed gently beneath the gum, then sawed upward from the gum over each tooth in turn. The contacts and the embrassures will be cleansed.

There is a use of floss which I think is new and useful.

The floss may be thrown on both sides of a single tooth, crossed and the ends pulled alternately. In narrow necked teeth, which are the ones chiefly attacked by pyorrhoea, this works the floss down to the gum and cleanses the entire circumference except perhaps the middle of the buccal cervix.

Here a wood point held in a universal porte, such as a bent brass tube, is very useful. It can be passed over every cervix except deeply between the teeth and there the floss does the work. Will the patient use it? Perhaps not, they may prefer to trust a patent tooth wash, powder or paste and then wonder why the teeth decay. I am reminded here of a remark made by Prof. Brubaker, "Mankind does not want to be dentally saved any more than it wants to be medically saved."

Less I be thought uniformed I wish to touch upon a thought that an idea has been promulgated that caries susceptibles have a carbohydrate in the saliva which acts as a food for the bacteria in the plaques or even assists in plaque formation and that future immunity lies in dietetics or a restrained carbohydrate diet, it being the thought that an excess in the diet causes an increase in glucose in the blood and thus in the saliva.

There is also the thought that mucin or aglutinin, or in other words the organic basis of the plaque, is of systemic and even mainly dietetic origin.

If these contentions are eventually proven there may be some help afforded patients, but even then the plaque is the local agent to be combatted in both caries and pyorrhoea. We know that clean teeth do not decay or have pyorrhoea—if kept clean, not kept unclean and called clean, and bacteriologically the difference is that between day and night.—*The Garretsonian.*

